# Supplementary material for: Allometric equations for estimating above-ground biomass of Nitraria sibirica Pall. in Gobi Desert of Mongolia
Source: PLoS One. 2020 Sep 29;15(9):e0239268. doi: 10.1371/journal.pone.0239268 (PMC7526795; doi:10.1371/journal.pone.0239268)
Supplement: Attachment — Submitted filename: Response to Reviewers.docx [file pone.0239268.s001.docx]

**Response to Editors**

Dear Academic Editor,

We would like to express our gratitude for your suggestions. They definitely improved and refined our manuscript. Our response to your comments/suggestions are as follows.

*Question 1: Please ensure that your manuscript meets PLOS ONE's style requirements, including those for file naming.*

Answer 1: We have double checked the requirements and our manuscript meets PLOS ONE's style requirements.

*Question 2: In your Methods section, please provide additional location information, including geographic coordinates for the data set if available.*

Answer 2: As you have suggested, we have provided additional location information such as geographic coordinates for the data set.

*Question 3: In your Methods section, please provide additional information regarding the permits you obtained for the work. Please ensure you have included the full name of the authority that approved the field site access and, if no permits were required, a brief statement explaining why.*

Answer 3: We have obtained permission to conduct field study in Great Gobi “A” Strictly Protected Area from Strictly Protected Area Authority of Ministry of Environment and Tourism, Mongolia. Permission document has been translated from Mongolian to English and scanned document is attached below.

Translation:

An official letter from

Ministry of Environment and Tourism,

Mongolia

“About issuing permission”

We have read through your request about conducting study in Strictly Protected Area. According to an Article 27.2 of the Law on Strictly Protected Areas, we grant permission to study the community structure of 4 important dietary plant species of Gobi bear and collect soil samples in Great Gobi “A” Strictly Protected Area. In the course of the field survey, the relevant provisions of Article 41 of the Law on Strictly Protected Areas should be implemented, and the field survey should be carried out under the supervision of the Great Gobi Strictly Protected Area “A”.

Signed and sealed by the Head of the Strictly Protected Area Authority, Mr. Sansarbayar E.

*Question 4: We suggest you thoroughly copyedit your manuscript for language usage, spelling, and grammar. If you do not know anyone who can help you do this, you may wish to consider employing a professional scientific editing service.*

Answer 4: We have copyedited our manuscript for any grammatical mistakes, spelling and language usage. Dr. Brandon Bestelmeyer, USDA-ARS Range Management Research Unit, Jornada Experimental Range has edited our manuscript.

*Question 5:We note that Figure 1 in your submission contain [map/satellite] images which may be copyrighted. All PLOS content is published under the Creative Commons Attribution License (CC BY 4.0), which means that the manuscript, images, and Supporting Information files will be freely available online, and any third party is permitted to access, download, copy, distribute, and use these materials in any way, even commercially, with proper attribution. For these reasons, we cannot publish previously copyrighted maps or satellite images created using proprietary data, such as Google software (Google Maps, Street View, and Earth). For more information, see our copyright guidelines:* [*http://journals.plos.org/plosone/s/licenses-and-copyright*](http://journals.plos.org/plosone/s/licenses-and-copyright)*.*

Answer 5: Figure 1 in our manuscript is not copyrighted. The map in Figure 1 was created by our co-author Munkh-Erdene Batsaikhan. Shapefiles of aimag, soum boundary and Great Gobi “A” Strictly Protected Area have been downloaded from an Open Source: Mongolian Databases of Environment and Administration of Land Affairs, Geodesy and Cartography.

*Question 6: We note you have included a table to which you do not refer in the text of your manuscript. Please ensure that you refer to Table 3 in your text; if accepted, production will need this reference to link the reader to the Table.*

Answer 6: As you have suggested, we have referred Table 4 (due to the addition of new table, Table 3 is now Table 4) in the text of our manuscript.

**Response to Reviewers**

Dear Reviewers,

Thank you so much for your comments. We appreciate all of your comments and suggestions which improved the quality of our manuscript. Our response to your comments/suggestions are as follows.

**Reviewer #1**

*Question 1: Strict English checking from a native speaker is important. MS lines were not included and hence hard to mention the proper places for revision. Please replace “ours were” by “our study was” or “this study was”. Fig. 3 replace “our equation” by “this study”. Major revision needed in English.*

Answer 1: Thank you for going through and pointing out the grammatical mistakes in detailed way. We have received help from an English expert and edited our manuscript.

*Question 2: Abstract: Research aim and conclusions are not very attractive. I suggest to write based on knowledge gap, and what you have contributed to ecology.*

Answer 2: We have revised our abstract as you have suggested.

*Question 3: Introduction: Very short paragraphs included. I suggest to merge small paragraphs in to total of three paragraphs including the last one as it is.*

Answer3: As you have suggested, we have merged small paragraphs into total of three paragraphs.

*Question 4: M&M section is generally nice, but try to improve if you can.*

Answer 4: Material and methods section has been revised as well.

*Question 5: Results: Table 2 can be merged in Table 1 by highlighting the best equations in bold color with gray background. Write some details in the caption what represents the best equation.
Fig. 3 is not very clear to me. Please use different relatively different colors for symbols and also for regression lines. Hard to assess which equation is over or under estimating the biomass. However, I think that Yang et al. is under estimating the biomass whereas your equation is over estimating the biomass. So, Ali et al and Conti et al are doing good as they the dots are near to the line but bit over estimated as your equation. Have a look carefully.*

Answer 5: The best equations have been highlighted in bold and details about best equations have been written in Table 3. We have omitted Figure 3 as the figure was not very clear, instead we have included the results as a text in the manuscript.

*Question 6: Discussion. Major revision is needed here. What is the contribution of your study to allometric biomass equations and ecology in general? Cite more relevant papers. Go beyond the simple discussion. Say something broad. You have compared results with three studies but those studies have recommended equations for species-specific and multispecies equations. I think, you are comparing the multispecies equations with your one species equation. So, there must be some differences. For example, in discussion you mentioned that your results are different than Ali et al where they found D, H and wood density as best predictors. So, you need to show further explanation, why? Same for other two studies (yang et al. and conti et al.). I am confused that why you are not comparing models results with Conti et al. 2015 (Ann For Sci) which was conducted in semi-arid regions.*

Answer 6: As there was no literature about biomass equation on our study object -*N.sibirica* Pall., we have compared our species-specific model with multispecies model from other authors. The application of multispecies model is broad and can be used for multiple species. Therefore, we have selected journals which have developed multiple species model and compared our results. As you have suggested, we have compared our model results with Conti et al. 2015 and showed further explanation on our comparison results with other papers.Consequently, Discussion part has been revised and edited.

**Reviewer #2**

*Question 1: Biomass is an important component of global terrestrial ecosystem carbon stocks. Here, the author developed a set of statistical models to estimate above-ground biomass of N. sibirica. The results will improve our predictive ability in aboveground biomass. However, the hypothesis, objective and rationale are not clearly presented. For example, the author claimed that grasses destructive harvest is difficult by citing Hughes et al., 1999. However, in Hughes’s paper, there is no mention of the difficulties in grasses destructive harvest.*

Answer 1: As you have suggested, we have re-edited our manuscript. Also, we have edited our citation by citing Louhaichi et al., 2018. in a sentence: “… in which destructive harvest is time-consuming and costly[3].”

*Question 2: In addition, I have some suggestions for revision: In the Material and methods section, all the field data are used to fit the allometric model. Generally, in order to verify the power of the statistical model, I suggest that you can use some data (eg. 75%) to estimate parameters and the rest of data (eg. 25%) to verify the model.*

Answer 2:Thank you for your suggestion. We have used some of our data to estimate parameters and other data to verify the model previously.

*Question 3: In Figure 3, the x and y axis are all ranged from 0 to 25. But why is the 1:1 line (intercept = 0) not a diagonal?*

Answer 3:We have omitted Figure 3 as the figure was not very clear, instead we have included the results as a text in the manuscript.
